# Wavelet-based assessment of postural stability in young female volleyball players using a baroresistive platform

**DOI:** 10.1371/journal.pone.0355995

**Published:** 2026-08-20

**Authors:** Marcin Śliwa, Adam Czaplicki, Tomasz Sacewicz

**Affiliations:** Faculty of Physical Education and Sport in Biała Podlaska, Józef Piłsudski University of Physical Education in Warsaw; Opole University of Technology: Politechnika Opolska, POLAND

## Abstract

The primary aim of this study was to evaluate the postural stability control mechanisms of young female volleyball players using wavelet analysis. The practical objective of the study was also to validate the signal derived from a baroresistive platform. The study involved 93 young female volleyball players (age: 15 years; height: 174.1 cm; body mass: 62.7 kg) and 30 female university students (age: 20.3 ± 0.6 years; height: 168 ± 3.5 cm; body mass: 61.9 ± 5.4 kg). All participants performed a standard 30-second Romberg test on an instrumented treadmill (Noraxon FDM-T) under both eyes-open and eyes-closed conditions. A time–frequency analysis of the treadmill signal was conducted using the Morse wavelet. The obtained results revealed several aspects of postural stability control in young volleyball players that may be the consequence of long-term athletic training. The treadmill signal demonstrated discriminative properties within the frequency ranges associated with visual and vestibular control.

## Introduction

Postural stability is one of the key components of motor ability that determines movement efficiency across many sports [1,2], including volleyball [3]. The ability to maintain a stable upright position relies on the integration of sensory inputs-proprioceptive, vestibular, and visual—and their central processing to generate appropriate motor responses [4]. These processes must account for various internal constraints and changing environmental conditions (e.g., the dynamic nature of gameplay).

Theoretical models of postural stability explain the general principles of balance maintenance; however, a complete understanding of postural mechanisms is still lacking, particularly regarding their dependence on age, level of athletic proficiency, and the specific demands of different sports [3,5,6].

In volleyball, as a dynamic team sport, postural stability control plays a crucial role in the effective execution of technical skills such as passing, serving, attacking, and blocking. Players must constantly adjust their body positions in response to rapidly changing on-court situations, which requires high precision, quick reaction time, and efficient balance management. The typical “ready position” in volleyball-characterized by a lowered center of gravity and a wide base of support-provides the foundation for anticipation and rapid movements essential for effective response to the opponent’s actions.

Research on postural stability control in sports indicates that athletes across different sports develop sport-specific postural strategies in response to the unique demands of their activity. For instance, shooters [7] and golfers exhibit exceptionally low body sway velocity [8], reflecting the necessity to maintain nearly static body configurations during precise movements. In team sports such as volleyball, postural stability is more dynamic and relies on the ability to rapidly adapt body position to the continuously changing context of the game [9,10].

The literature also includes studies examining postural control in dancers [11], gymnasts [12], football players [13], and volleyball players [9]. In certain sports, training may modify the influence of visual stimuli on balance control. Dynamic posturography research indicates that professional male dancers exhibit significantly greater stability and less visual dependence in postural control than untrained individuals [14]. The authors suggested that professional dance training enhances proprioceptive accuracy and shifts sensorimotor dominance from vision to proprioception.

Volleyball players maintain continuous visual engagement during gameplay. Observing players’ eye positions during defensive actions reveals that proper ocular function is fundamental to effective response to the opponent’s attack. The constant motion of the ball across the court requires intensive activity of the ocular muscles, which may, in turn, influence postural control mechanisms. Numerous studies have demonstrated associations between athletes’ performance in specific sports and their visual abilities [15,16].

A study conducted on male volleyball players showed that experienced athletes exhibit fewer but longer fixations, focusing on the initial and final points of the ball’s trajectory, whereas non-athletes tend to follow the entire trajectory [16]. Rougier and Garin [17] found that saccadic eye movements can modify postural control during quiet standing. In the present study, we investigated body sway during a bipedal standing task in volleyball players and non-athletes, as well as the influence of the visual system on postural control.

Starting with Hellebrandt's pioneering work [18], basic tests used to assess body posture stability have been continuously based on the study of center of pressure (COP) kinematics [19]. Much work has also been devoted to spectral analysis of COP time series [20–25]. Although individual researchers have focused on different frequency bands of the power spectrum, it is believed that frequencies below 0.1 Hz may be related to visual control, frequencies in the range <0.1; 0.5> with vestibular control, while higher frequencies up to about 1 Hz are attributed to sensorimotor control [26–28]. It has been shown that about 90% of the signal energy in the anterior-posterior direction is contained in the frequency band up to 2 Hz [21]. It has also been noted that COP time series are non-stationary in nature [29,30], which has led to the use of wavelet analysis in the study of these waveforms [31].

COP time trajectories are calculated based on ground reaction forces measured by force platforms. Regardless of how the instantaneous position of the COP is determined, the coordinates of this point depend mainly on the vertical reaction values measured by individual force sensors (e.g., BioWare manual). It therefore seems somewhat surprising that spectral analyses of the time courses of these reactions are conducted only sporadically. Despite the existence of such studies [32–36], the power spectrum of ground reaction forces does not have as clear an interpretation as the COP spectrum. An additional argument in favor of studying the spectrum of vertical ground reaction is the rapidly growing use of devices equipped with sensor arrays that measure foot pressure on the ground, such as treadmills, baropodometric platforms and foot pressure plates. These devices generate an output signal in the form of time waveforms of the ground reaction to different areas of the foot, and the spectral analysis of such a signal has not yet been fully explained.

The aim of this study is to identify a way to assess body posture stability in young female volleyball players during bipedal stance on a platform equipped with a baroresistive sensor matrix. The practical aim of the study was also to verify the usefulness of spectral analysis of signals from this type of platform.

## Materials and methods

### Participants

The study included 93 female volleyball players aged 15 years (height: 174.1 cm; body mass: 62.7 kg) who participated in the Olympic Hopes Tournament (OHT). All participants reported being enrolled in sports-oriented classes specializing in volleyball. Their weekly training volume consisted of four 90-minute specialized volleyball training sessions and four 45-minute physical education classes conducted in accordance with the core curriculum of the Ministry of Education and Science.

Parents or legal guardians provided written informed consent for their child’s participation in the study. Both the participants and their parents/guardians were informed about the research procedures, as well as the potential risks and benefits associated with participation in the project.

The study also involved 30 female university students (age: 20.3 ± 0.6 years; body mass: 61.9 ± 5.4 kg; height: 168 ± 3.5 cm). The research protocol was approved by the Senate Ethics Committee of the Józef Piłsudski University of Physical Education in Warsaw (SKE 01–15/2023) and was conducted in accordance with the principles of the Declaration of Helsinki. The measurements were conducted at the Regional Center for Research and Development, University of Physical Education Branch in Biała Podlaska, in the Biomechanics and Kinesiology Laboratory.

### Measurements

The participants performed a 30-second standard Romberg test with arms extended horizontally while standing on a treadmill (Noraxon FDM-T) equipped with a platform with an integrated baroresistive sensor array (5,376 sensors, density of 1.4 sensors/cm^2^, measurement range of up to 120 N/cm^2^). The sampling frequency of the platform was 100 Hz. The Romberg test was conducted in two modes. In the first mode, the subjects had their eyes open, while in the second mode, they had their eyes closed. Prior to the test, the participants performed a 20-second free standing test on a treadmill to adjust to the non-standard surface and body configuration required for this study.

### Data processing

**Fig 1:**
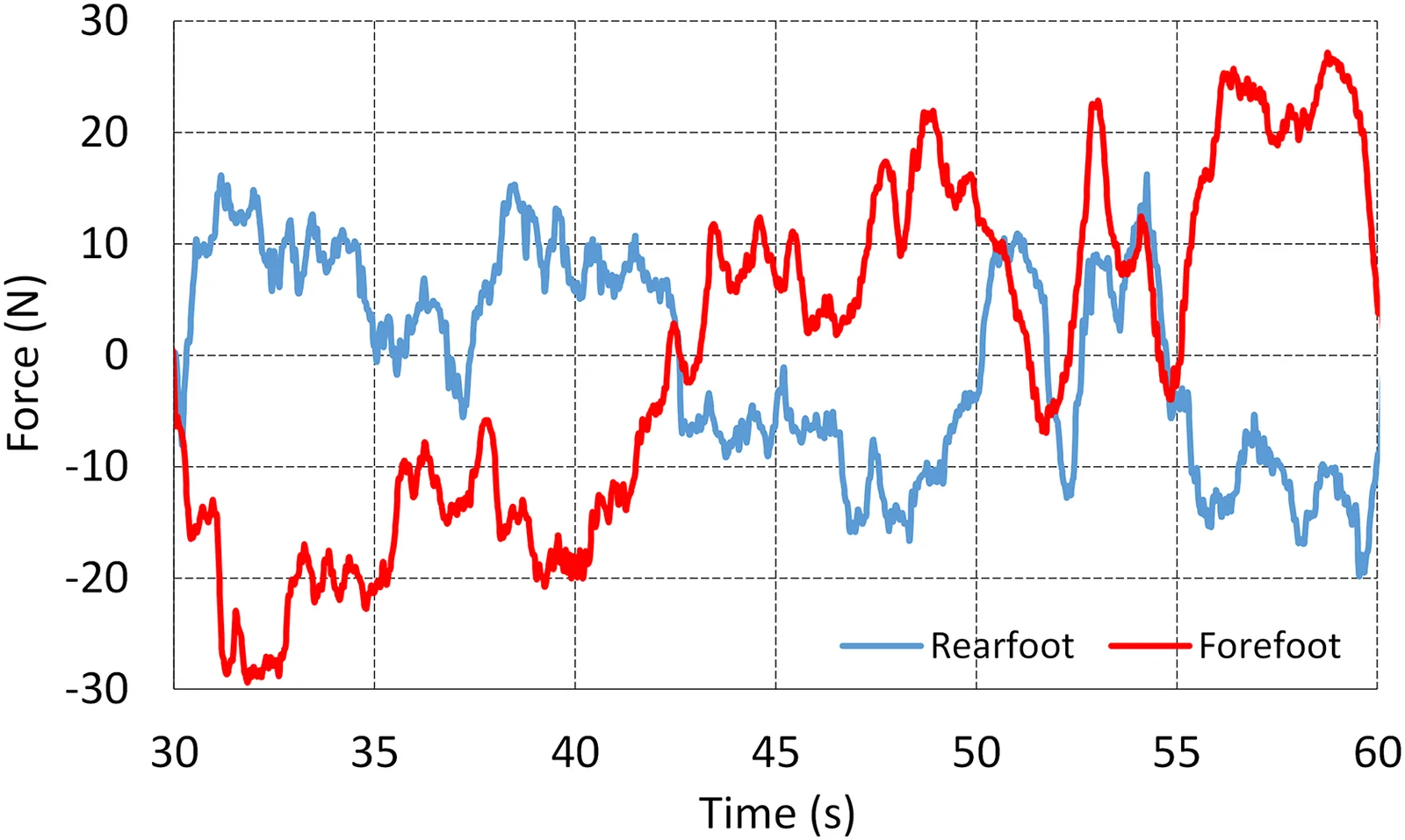
Pre-processed time courses of vertical ground reaction force of one of the volleyball players in open eyes mode.

The software provided by the treadmill manufacturer allows the time trajectories of the resultant vertical ground reaction force under the forefoot and rearfoot to be obtained. This signal was zeroed to the mean value and smoothed with a low-pass filter with a cut-off frequency of 10 Hz. The preprocessed ground reaction time waveforms under the forefoot and rearfoot were found to be largely symmetrical about the abscissa in all participants (Fig 1).

The vertical reaction force time trajectories for the rear of the right foot (VGRRF -Vertical Ground Reaction for the Rear of the Foot) were therefore selected for further analysis. These were symmetrically, bilaterally zero-padded to a total length of 9,000 points, ensuring that a minimum frequency of *f* = 0.039 Hz was achieved for the lowest-scale wavelet.

It should be clearly emphasized here that the VGRRF analyzed in this study corresponds to approximately 25% of total body weight. This is because about 55% of this weight is absorbed by the rearfoot [37], and a slight change in the position of the body's center of gravity with the upper limbs extended horizontally to the ground is about 5%, which has been estimated based on commonly available geometric and mass data [38].

**Fig 2:**
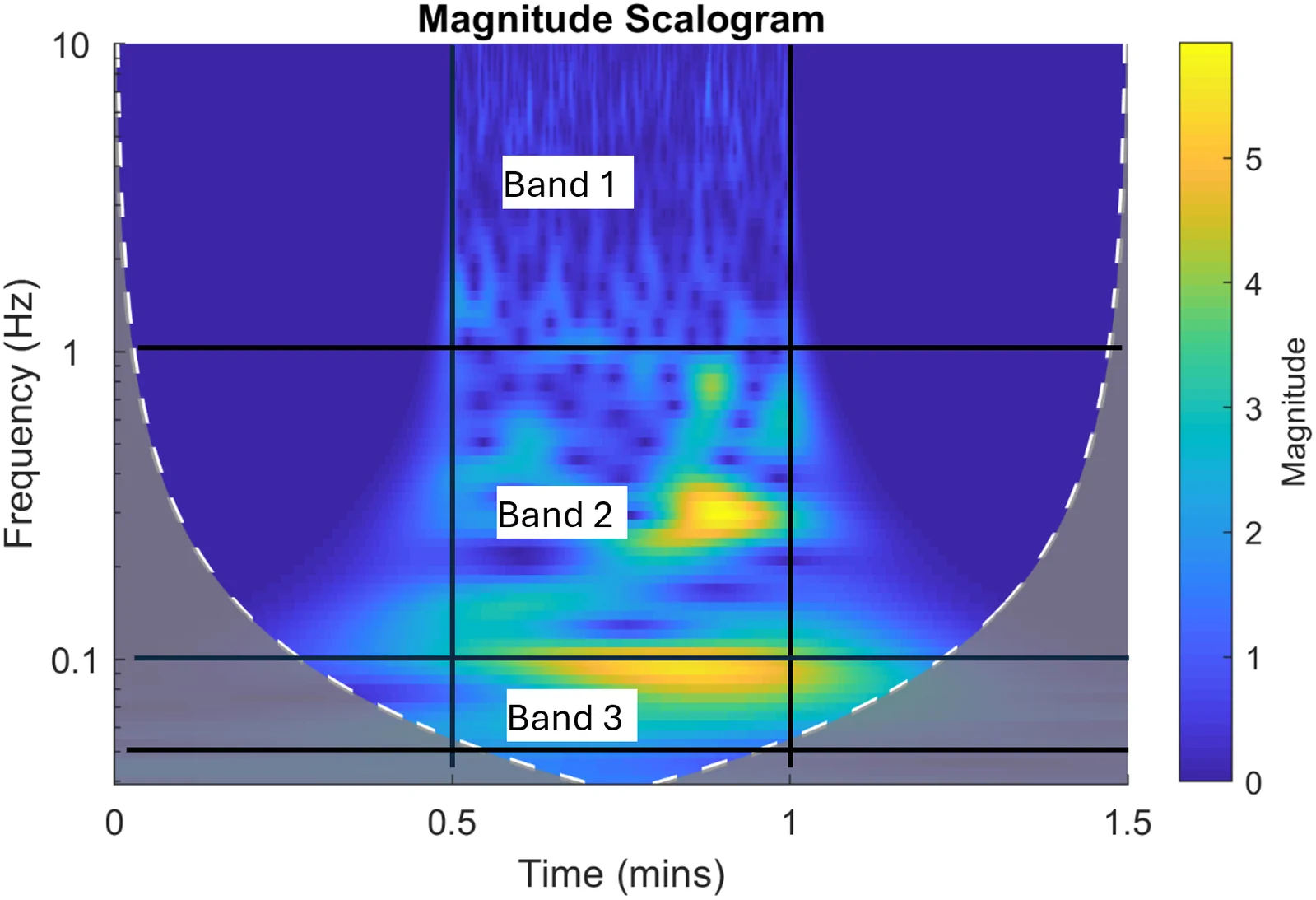
Scalogram of the time course of VGRRF, as shown in the Fig 1.

The wavelet analysis was performed using Morse wavelets (Matlab Wavelet Toolbox, MathWorks, USA). To reduce boundary effects when calculating signal energy, a rectangular region <30 ÷ 60 s > ∩ < 0.05 ÷ 10 Hz > was extracted from the time-frequency plane inside the influence cone (Fig 2). The total energy of the transformed signal in this area, the energies contained in the three frequency bands 0.05 < *f* ≤ 0.1, 0.1 < *f* ≤ 1 and 1 ≤ *f* < 10 and the ratios of these energies to the total energy were then calculated.

**Fig 3:**
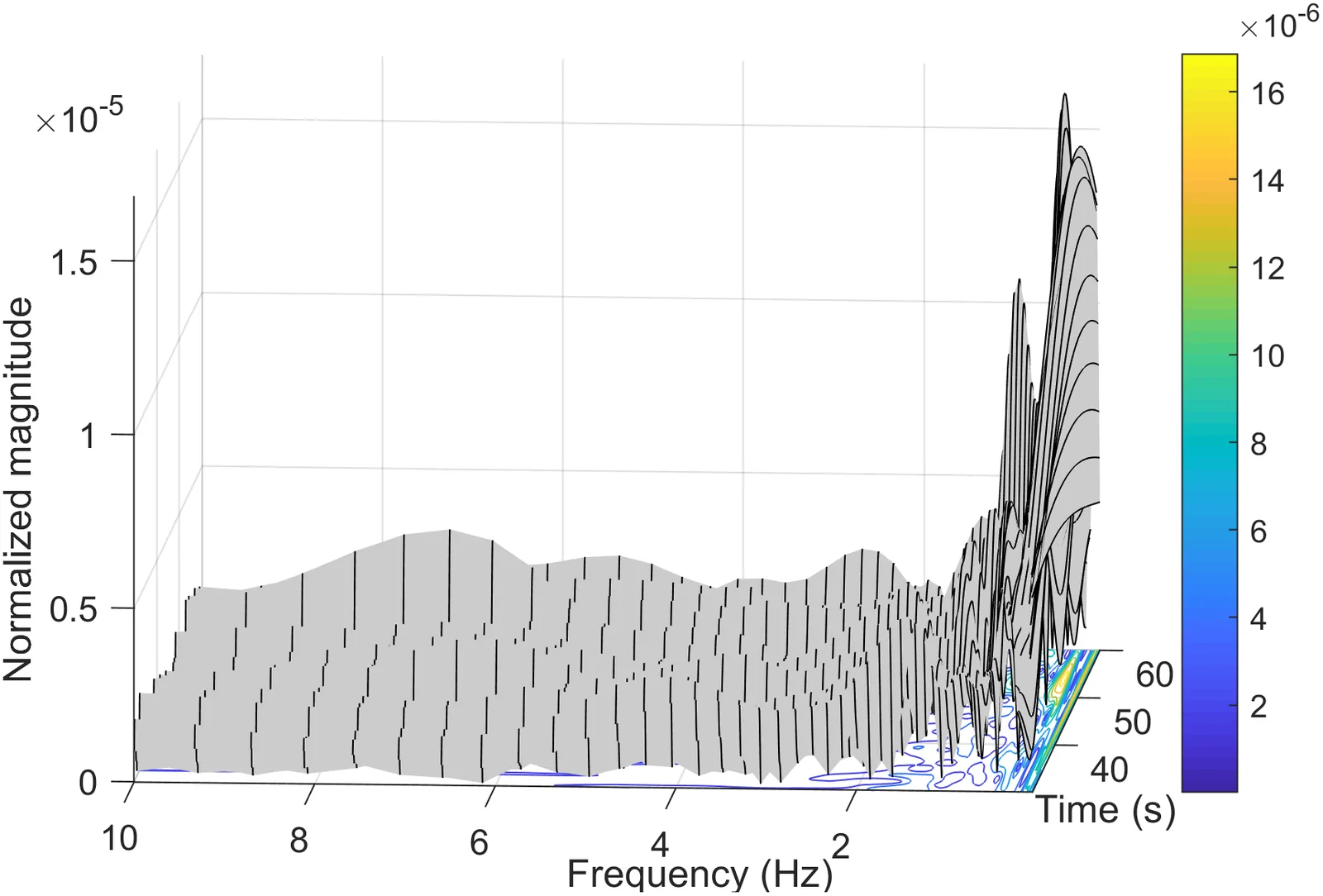
Normalized time-frequency distribution of relative energy corresponding to the scalogram in Fig 2.

To identify the location of VGRRF in time, we used time-frequency density plots, which are directly related to the energy distribution of the transformed signal. The graphs of each test subject were first normalized to the sum of the modules of all wavelet coefficients (Fig 3). Next, the positions of the centroids of the normalized coefficients were determined in three rectangular areas labelled Band 1, Band 2 and Band 3 (Fig 2). Finally, the positions of the resultant centroids were calculated for all subjects in a given group and for the trial mode.

### Statistical analysis

A regression approach was used in the analysis of energy distribution in each frequency band. The calculations were performed in the R environment (R Foundation for Statistical Computing, Austria) using the *lme* function from the *nlme* package [39]. We defined two linear mixed models with repeated measurements. In the first model, we specified energy distribution within individual bands as the dependent variable, and exercise mode (open vs. closed) and group (volleyball players vs. students) as categorical variables. The second model evaluated differences in energy distribution patterns across exercise modes and study groups. The group variable was substituted with a categorical factor representing successive measurements of the dependent variable within the selected energy distribution bands, capturing within-participant variability. To account for individual variation, different random intercepts were assigned to the participants as the random part of both models.

Pairwise comparisons were performed using the *emmeans* package in R, applying a Bonferroni correction for multiple comparisons, to examine differences between mean values.

When reporting effect magnitudes, we calculated standardized effect sizes from the Cohen’s *d* family, given the hierarchical structure of mixed-effects modeling.

## Results

Table 1 shows the results of the statistical analysis of the recorded signal, expressed as a percentage of total energy. Significant differences were found in the first and in the second frequency band between the results of the tests with eyes open and closed for both groups.

**Table 1:** Percentage distribution of the total energy of the transformed signal (S1 File).

	Band 1	Band 2	Band 3
Volleyball players eyes open	26.25 ± 13.68^○^	65.78 ± 13.52^+^	7.98 ± 6.95
Volleyball players eyes closed	21.94 ± 11.98^○^	70.28 ± 11.44^+^	7.78 ± 5.32
Students eyes open	29.73 ± 13.31^•^	61.91 ± 12.56	8.36 ± 4.73
Students eyes closed	18.04 ± 7.60^•^	73.57 ± 8.27	8.39 ± 5.13

Identical symbols indicate statistically significant differences, ^○^ *p* ≤ 0.0197, *d* = −0.394; ^•^ *p* ≤ 0.0001, *d* = −1.017;^+^ *p* ≤ 0.0122, *d* = 0.414; *p* ≤ 0.0001, *d* = 1.016.

**Fig 4:**
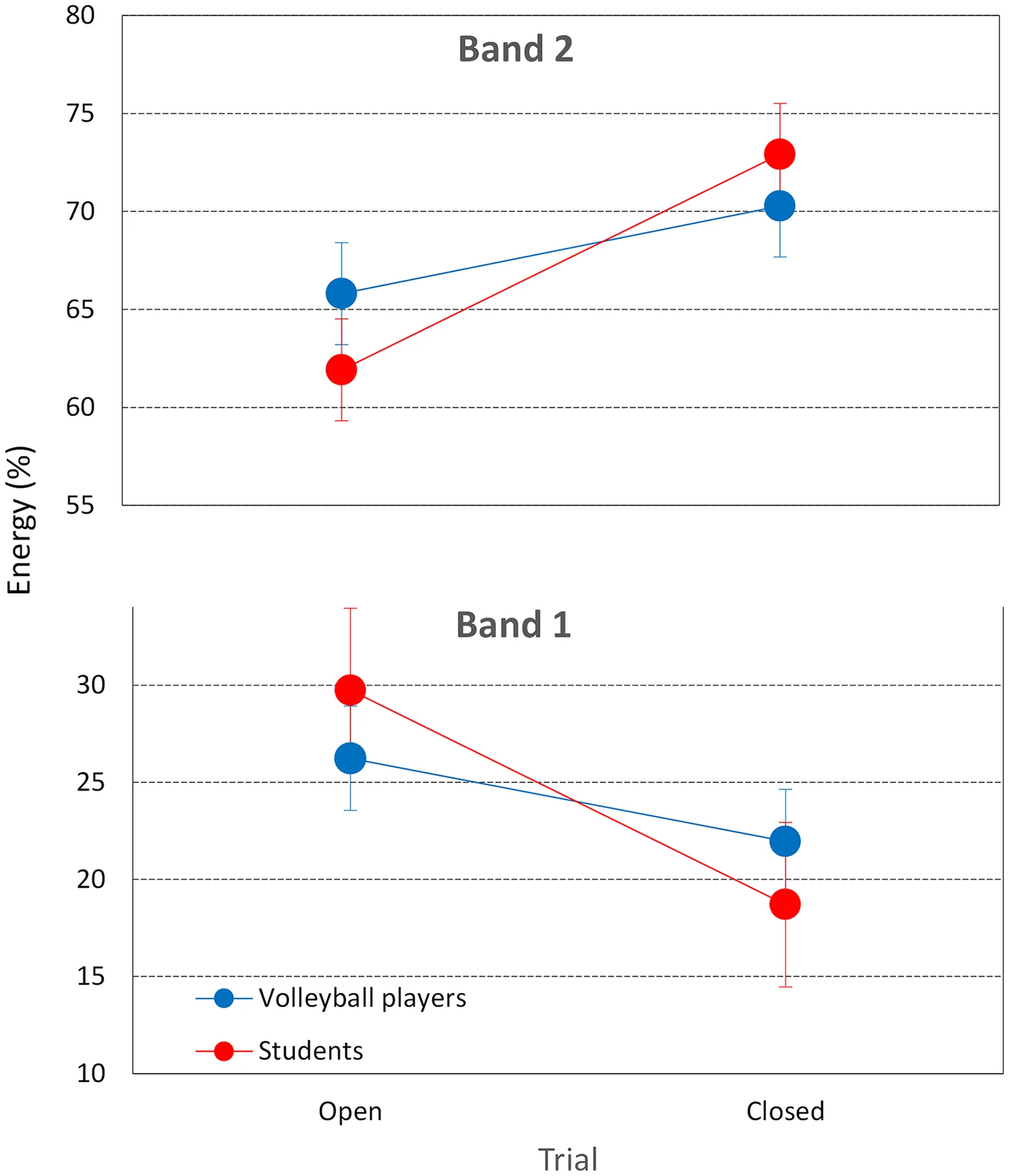
Interaction effect of group and trial type on energy levels.

Alongside the main effects presented in Table 1, post-hoc decomposition of the interaction regarding the percentage distribution of energy in Band 1 indicated that moving from the *open* to the *closed* trial led to a significant decrease in this band's energy share for both groups (p ≤ 0.045). The magnitude of this change depended significantly on the group. The energy share in Band 1 decreased by 11.02% in the student group, whereas volleyball players group exhibited a substantially smaller decrease of 4.27% (Fig 4, bottom).

Regarding the second frequency band (Band 2), the linear mixed-effects model revealed a marginally significant interaction effect between group and trial type on the percentage distribution of energy (p ≤ 0.051). The energy share in Band 2 increased by 11.00% in students’ group, whereas volleyball players group exhibited a substantially smaller increase of 4.48% (Fig 4, top).

When presenting the regression analysis results, it is worth emphasizing that the underlying assumptions of the method were fully met, including the homoscedasticity of residuals (*p* = 0.976).

**Fig 5:**
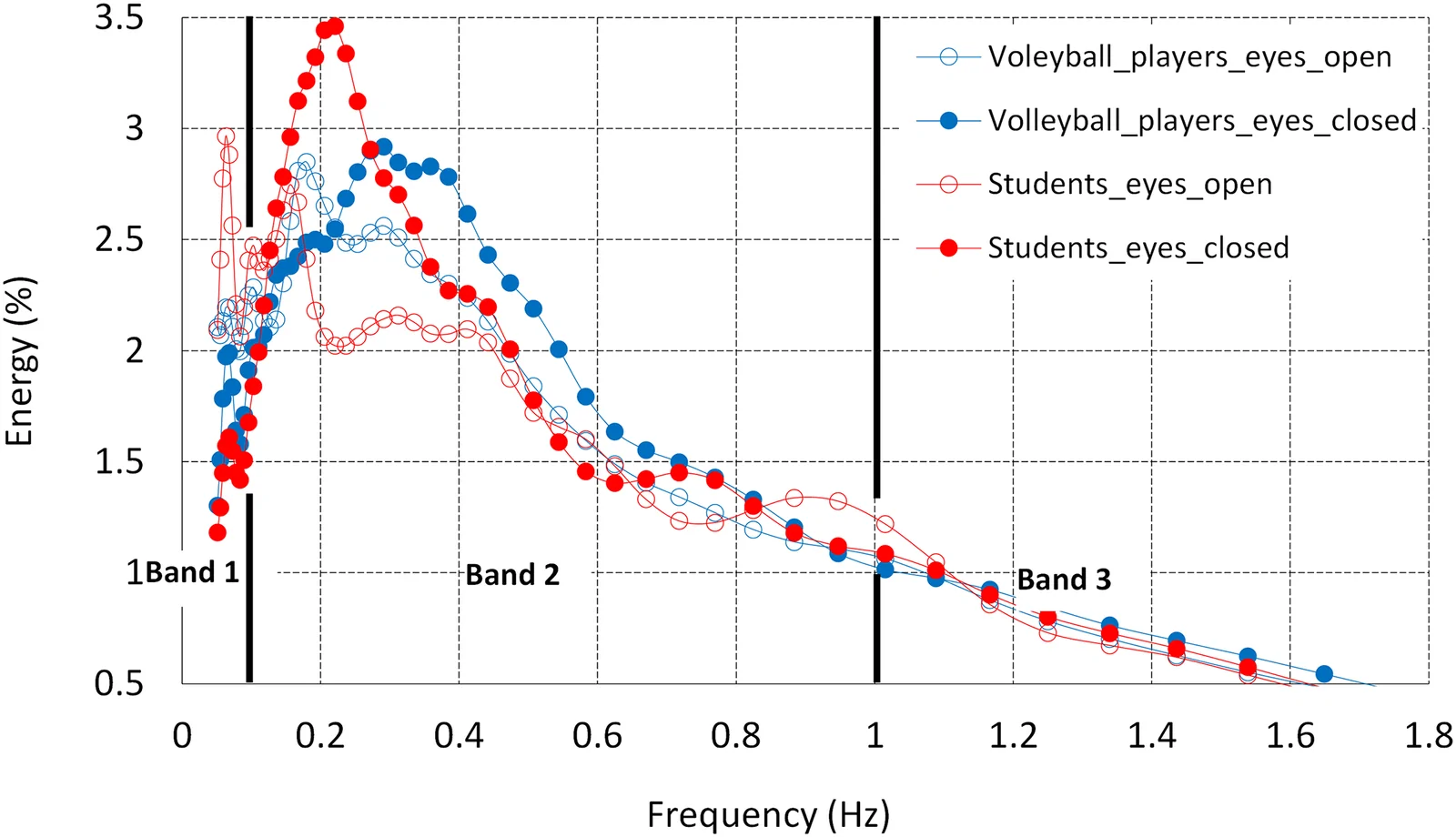
Average percentage distribution of signal energy from the baroresistive platform.

Fig 5 presents the average percentage energy distribution curves in the three analyzed frequency bands. Peaks in the relative energy of the transformed signal are visible in the volleyball players’ group for frequencies around 0.18 Hz (eyes open) and 0.29 Hz (eyes closed) and in the student group for frequencies 0.06 Hz and 0.17 Hz (eyes open) and 0.22 Hz (eyes closed). In the student group, the second linear mixed model revealed statistically significant differences in energy values between the open-eye and closed-eye conditions within the 0.05–0.07 Hz and 0.19–0.27 Hz frequency bands. For instance, for the relative energy peak in the first mentioned band, the difference was significant at *p* ≤ 0.0002 (*d* = 1.04), and for the peak in the second band at *p* < 0.0001 (*d* = −1.34). In the case of volleyball players, we did not observe such differences in the distribution between the two trials.

**Fig 6:**
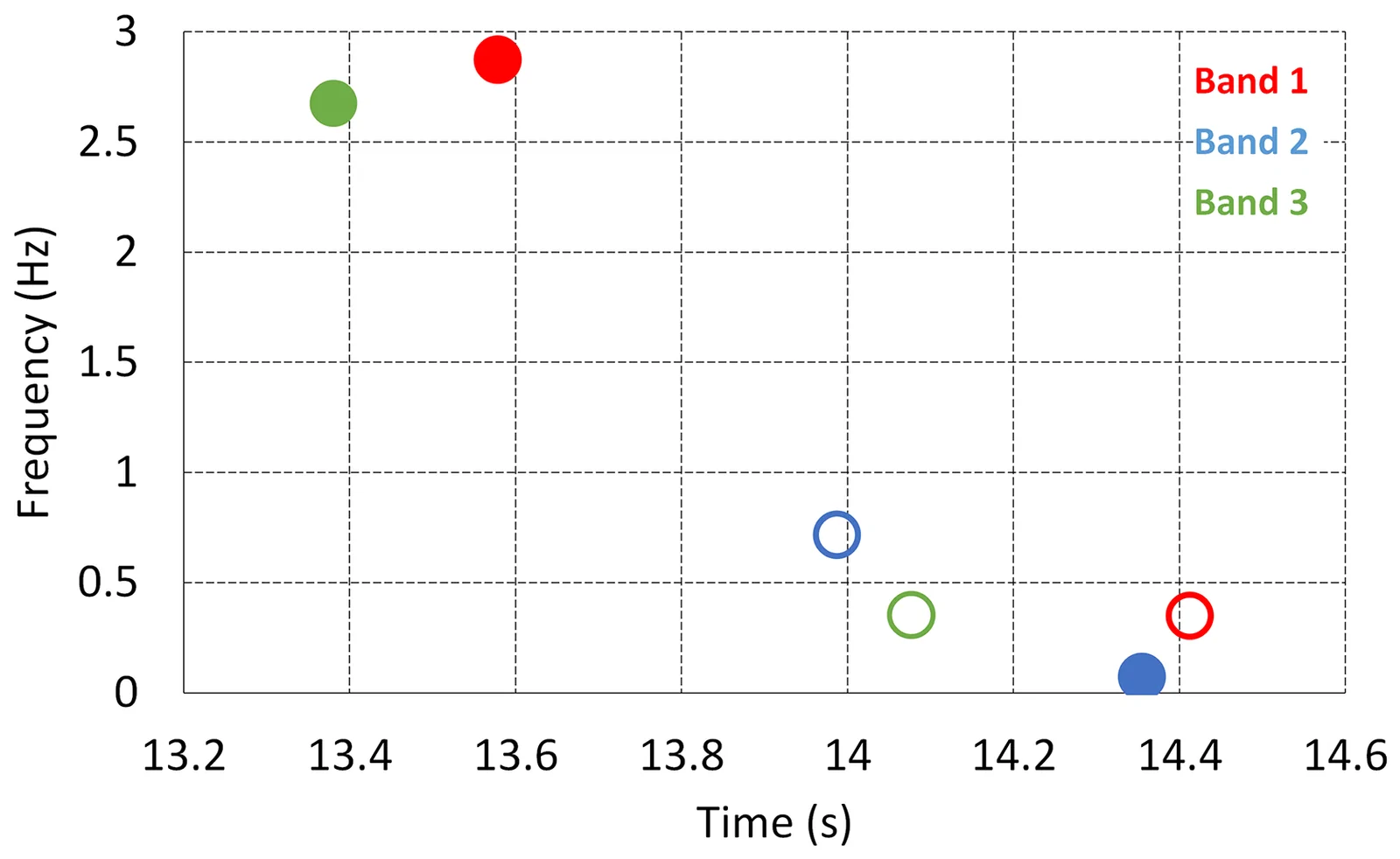
Location of resultant modules in the time-frequency plane for volleyball players.

Fig 6 shows the positions of the resulting modules in the time-frequency plane for tests with eyes open (open dots) and closed (closed dots) for the three areas studied. Only the difference between the times for Band 1 was statistically significant (p < 0.015).

## Discussion

One of the main objectives of the study was to analyze the control of body postural stability in young volleyball players during the Romberg test on a baroresistive platform. In both groups, we observed a statistically significant decrease in the average energy level in the first frequency band coupled with an increase in energy in the second band between the two trials. The results obtained thus confirmed the generally known fact of a shift in the maximum values of the transformed signal spectrum towards higher frequencies in the case of the test with closed eyes [14,27]. The value of this shift was similar in both groups, but the control of the volleyball players’ posture depended to a much lesser extent on visual stimulation. This is because post-hoc decomposition of the interactions revealed distinct dynamics between the two groups when transitioning from the open to the closed trial. Concerning the energy share in Band 1, closing the eyes led to a decrease across both groups, but the response in volleyball players group was significantly attenuated. A mirror pattern of reactivity was captured in Band 2, where moving to the closed condition triggered an increase in the energy share for both groups, which was notably buffered in volleyball players group. This described energy transfer is also visible in Fig 5, which further confirms the activation of vestibular control channels [26,40].

The obtained results suggest that this kind of energy transfer in volleyball players may be a consequence of long-term training, which involves numerous situations requiring the maintenance of balance under dynamic conditions—such as during jumps, landings, sudden changes of movement direction, or defensive actions. In such moments, players must react instantaneously to visual stimuli while maintaining body stability despite limited visual control, for example when their gaze is focused on the ball above their heads. Continuous repetition of these types of actions develops in players the ability for automatic postural control and more effective use of information derived from the proprioceptive and vestibular systems. As a result, volleyball players rely less on visual analysis when maintaining balance, which translates into greater stability and precision of reactions in dynamic match situations.

Our findings are also consistent with previous reports indicating that long-term sports training enhances the integration of sensory signals and increases the contribution of proprioceptive and vestibular control [3,6].

Based on the results of the time-frequency analysis of the tested signal, we also found that there is a statistically significant difference in the time location of the resultant energy in the first frequency band between the test with eyes closed and eyes open in the group of volleyball players. In the latter case, this location is closer to the middle of the test duration. This means that volleyball players are able to evenly distribute their response to long-term oscillations over time. This ability can be transferred to the game, where there are natural waiting periods of up to several seconds for the opponent's serve. Therefore, from a coaching perspective, balance and postural control training should be implemented not only under dynamic conditions but also through tasks requiring prolonged maintenance of muscle tension and a stable ready position, as this promotes the development of a more efficient model of postural control.

The practical aim of the study was to validate the signal from the baroresistive platform. The results showed that, regardless of the test mode, approximately 97% of the energy of the transformed signal is in the frequency band below 2 Hz. However, the results of the COP spectrum analysis in the anterior-posterior plane in people maintaining a standing position suggest that approximately 90% of this energy is in the band below 2 Hz [21]. Our calculations also show that approximately 78% of the energy is in the frequency band up to 0.5 Hz. This result is 10% higher than the results presented in [41], in which a short-term Fourier transform was used to determine the percentage distribution of COP sway energy in three frequency bands (0; 0.5 > , (0.5; 2> and >2 Hz) in the anterior-posterior plane in a group of regional-level judo competitors.

The energy distribution of the transformed signal turned out to be similar to the three-dimensional plots of COP sway in the time-frequency domain presented in the paper [30], although it lacks energy in the frequency band around 0.5 Hz [26].

The VGRRF spectrum also differs in shape from the spectrum of vertical ground reactions measured using traditional force platforms [32,42]. It also does not have peaks at frequencies around 4–6 Hz characteristic of that spectrum.

In summary, VGRRF analysis gives results similar to those of COP analysis during free standing compared to the results of ground reaction analysis, but for the reasons listed above, it should rather be treated as a new way of assessing balance control.

When discussing our results, at least three limitations that may have affected the reliability of the conclusions drawn should be mentioned.

Firstly, we arbitrarily selected the VGRFF for wavelet analysis. The analysis of plantar pressure distribution indicates that the rearfoot plays a fundamental role in maintaining postural stability during quiet standing. It has been demonstrated that, under static conditions, the center of pressure (COP) is typically located near the ankle joint, resulting in relatively greater loading of the heel region compared to other areas of the foot [43]. The importance of the rearfoot is further supported by correlation analyses, which show that plantar pressure parameters derived from the heel region exhibit stronger associations with global stability indices than those obtained from the forefoot or midfoot [44]. However, it should be noted that a comprehensive assessment of postural control requires consideration of both rearfoot and forefoot regions. While the present study focused on the rearfoot as the primary indicator of global stability, the forefoot plays a crucial role in the active regulation of balance, particularly through the execution of rapid corrective actions. This region has been shown to be associated with higher-frequency components of the COP signal, reflecting neuromuscular control processes and dynamic postural adjustments [45,46]. Therefore, exclusion of forefoot analysis may limit the ability to fully capture the complexity of postural control strategies, especially those related to fast, adaptive responses.

Secondly, the VGRRF values were calculated by the software of the FDM-T platform manufacturer (Noraxon, USA). Given the growing number of such measuring devices (pressure-instrumented treadmills, platforms and insoles) supplied by various manufacturers, it can be assumed that the algorithms for calculating VGRRF depend on the software supplied with the device. On the one hand, this limits the strength of the generalization of the conclusions drawn, but at the same time suggests further research in this direction.

Finally, the age of the control group differed from that of the volleyball players. However, previous studies indicate that postural control reaches a functional plateau in late adolescence, with parameters observed in adolescents closely approaching those of young adults. The largest differences in center of pressure (COP) characteristics are reported between children and adolescents, whereas further changes after approximately 15–18 years of age are relatively small or statistically non-significant [47,48] Additionally, it has been shown that under quiet standing conditions, differences between age groups may not be evident [49]. The results of studies of young volleyball players compared to their peers could also be important from a cognitive point of view**,** and we wanted to show the impact of many years of training on the posture control of young athletes compared to mature individuals.

## Conclusion

Wavelet analysis of the VGRRF signal revealed the impact of long-term training of young volleyball players on the control of their body stability in a standing position.

Since no significant spectral peaks were observed in the VGRRF spectrum beyond the 1 Hz threshold, transferring the conventional frequency band divisions used in COP trajectory analysis to VGRRF signals is methodologically unwarranted. Spectral investigations of VGRRF should utilize customized, lower-frequency sub-bands to accurately reflect its specific biomechanical energy distribution.

The use of pressure instrumented devices in body posture control research appears to be a useful research tool, especially in terms of the frequencies attributed to visual and vestibular control.

## Supporting information

S1 File Percentage energy distribution in three frequency bands. This spreadsheet contains the detailed calculation results for the energy distribution across the analyzed frequency bands.
(XLSX)
